# An Iterative Algorithm for Semisupervised Classification of Hotspots on Bone Scintigraphies of Patients with Prostate Cancer

**DOI:** 10.3390/jimaging7080148

**Published:** 2021-08-17

**Authors:** Laura Providência, Inês Domingues, João Santos

**Affiliations:** 1 Faculdade de Ciências, Universidade do Porto, 4169-007 Porto, Portugal; lauraprovid@hotmail.com; 2 Medical Physics, Radiobiology and Radiation Protection Group, IPO Porto Research Centre (CI-IPOP), 4200-072 Porto, Portugal; joao.santos@ipoporto.min-saude.pt; 3 Instituto de Ciência Biomédicas Abel Salazar, Rua de Jorge Viterbo Ferreira nº 228, 4050-313 Porto, Portugal

**Keywords:** bone scintigraphy, prostate cancer, machine learning, semisupervised classification, false positives reduction

## Abstract

Prostate cancer (PCa) is the second most diagnosed cancer in men. Patients with PCa often develop metastases, with more than 80% of this metastases occurring in bone. The most common imaging technique used for screening, diagnosis and follow-up of disease evolution is bone scintigraphy, due to its high sensitivity and widespread availability at nuclear medicine facilities. To date, the assessment of bone scans relies solely on the interpretation of an expert physician who visually assesses the scan. Besides this being a time consuming task, it is also subjective, as there is no absolute criteria neither to identify bone metastases neither to quantify them by a straightforward and universally accepted procedure. In this paper, a new algorithm for the false positives reduction of automatically detected hotspots in bone scintigraphy images is proposed. The motivation relies in the difficulty of building a fully annotated database. In this way, our algorithm is a semisupervised method that works in an iterative way. The ultimate goal is to provide the physician with a fast, precise and reliable tool to quantify bone scans and evaluate disease progression and response to treatment. The algorithm is tested in a set of bone scans manually labeled according to the patient’s medical record. The achieved classification sensitivity, specificity and false negative rate were 63%, 58% and 37%, respectively. Comparison with other state-of-the-art classification algorithms shows superiority of the proposed method.

## 1. Introduction

According to the World Health Organization, prostate cancer (PCa) is the second most commonly diagnosed cancer in men, accounting for more than 1.4 million new cases and more than 375,000 deaths worldwide in 2020. Patients with advanced prostate cancer often develop metastases, which are caused by primary tumor cells that escape from the prostate gland and spread through the lymphatic system or the bloodstream to other areas of the body. The most frequent site for metastatic growth of prostate cancer is the bone, and almost all patients with advanced prostate cancer show histological skeletal involvement, being estimated that 84% to 90% of patients with metastatic disease had bone metastases [1,2,3]. Even though the bone metastases are seldom the cause of death, they are the leading cause of morbidity and a major challenge in the management of patients, leading to a diminished quality of life. The presence of bone metastases, specially in higher extents, is an indicator of progression of the disease and typically correlates with a poor prognosis [4,5]. Currently there is no cure for metastatic prostate cancer, but it can often still be treated to slow down its growth. A precise detection and up-take quantification of bone metastases is essential to provide the physicians the accurate staging they require to choose the appropriate treatment for an individual patient, to monitor the evolution of the disease and to evaluate the treatment efficiency.

The most common diagnostic procedure used for screening, assessment of treatment and follow-up of patients with bone metastases is whole-body bone scintigraphy (BS) [6], due to its relatively high sensivity, ranging from 70% to 78% [7,8,9], and widespread availability at relatively low cost. Bone scintigraphy, also known as bone scan, is a nuclear medicine imaging technique used in screening for several skeleton related pathological conditions, including bone metastases. In a bone scintigraphy, a bone-seek radioisotope, that is, a substance that collects in the bones following the normal physiological processes, is injected intravenously into the patient. The radioactive isotope will flow through the body and will have a tendency to accumulate in areas of high bone metabolic activity. Following the radiopharmaceutical administration, a time period of 2 to 4 h [10] is observed to allow biodistribution and up-take and then a simultaneous image of the anterior (AP) and posterior (PA) views is acquired in a gamma-camera. Because the radioisotope has accumulated in the regions of bone, the scans will reveal brighter areas, which indicate an increased rate of bone metabolic activity such as abnormal growth caused by metastases. These areas are referred to as hotspots, and may indicate not only the presence of bone metastases, but also other conditions such as trauma, microarthritis, benign degeneration, or bone infections [11]. The biggest disadvantage in the use of bone scintigraphy to detect bone metastases is, therefore, its low specificity. Because it evaluates the distribution of active bone formation in the skeleton and identifies the sites where metabolic reactions are occurring, it detects several suspicious uptakes of nonmetastatic origin, which lead to high a false positive rate of BS to detect bone metastases. To date, the assessment of bone scans relies solely on the interpretation of an expert physician who visually assesses the scan. Besides this being a time consuming task, it is also extremely subjective, as there is no absolute and clear criteria neither to differentiate bone metastases from benign bone lesions, neither to quantity them. This means that, up to this date, the disease stage as well as the response to treatment is subjected to a certain degree of uncertainty, implying that the process of determining whether or not the patient condition is regressing is sometimes subjective. Given the high occurrence of metastatic PCa, there should be by now a more practical and, most importantly, more objective criteria to evaluate quantitatively a bone scintigraphy.

This work aims to create an algorithm capable of classifying hotspots from bone scintigraphy images, and is manly motivated by the call for a method whose development does not require a fully labeled database. A labeled data set of hotspots is rare and most likely unavailable for most researchers, and therefore one propose a semisupervised method that only requires knowledge about the type of bone scan the hotspot is extracted from. Comparison with other state-of-the-art classification algorithms shows superiority of the proposed method, achieving a sensitivity of 0.63, a specificity of 0.58 and a false negative rate of 0.37. This algorithm was able to decrease the false positive rate from 0.73 after detection to almost half, 0.42 after the false positive attenuation.

The main contributions of the present work include:

- The proposal of a new, iterative, semisupervised algorithm for attenuation of false positive metastases;
- Extensive experiments on a real dataset of scintigraphies from 102 patients with prostate cancer;
- A suggestion for a hotspots detection technique;
- Comparison with nonsupervised and one-class classifiers.

The remaining of this paper is organised as follows: Section 2 reviews the state of the art; Section 3 gives a detailed description of the here proposed semisupervised iterative algorithm; Section 4 gives the materials and methods, including the database, the hotspots detection technique, the extracted features, the competing classification algorithms for false positives reduction and the evaluation methodology; Section 5 presents the results, and Section 6 presents a discussion of these results. The document finishes with some conclusions and directions for future work in Section 7.

## 2. Related Work

The literature found on this topic shows there has been some effort to develop a computer-aided diagnosis system capable of automatically detecting and quantifying bone metastases in bone scintigraphies.

Brown et al. [12] developed a computer-aided system to automatically segment and quantify bone scan lesions. The bone lesion segmentation was accomplished by doing an atlas-based anatomic segmentation to divide the body into 6 different regions, followed by the application of region specific threshold to detect the hotspots. The method achieved a median sensitivity of 94.1%, specificity of 89.2% and accuracy of 89.4%. After the detection of the hotspots, the resulting images were reviewed by a nuclear medicine physician who removed false positive lesions; the hotspots classified as malign could then be used to assess the severity of the disease and disease response to treatment. Despite the good results, this algorithm is not fully automatic, as it requires the intervention of a physician to remove false positives (nonmetastases related bone uptakes) from the scans. This is a huge downside as the automatic differentiation between malignant and nonmalignant bone uptakes is an essential requirement in a bone metastases evaluation algorithm, as it is a task that is not trivial even for the most experienced physician and thus brings a lot of subjectivity to the final assessment. A classification algorithm capable of automatically distinguish metastases from benign lesion is thus needed.

Sadik et al.  [13,14] developed a fully automated classification system for the detection of metastases that used artificial neural networks. Both works intended to classify the whole-body bone scan as a whole, regarding the presence or absence of bone metastases, and not the hotspots individually. The final classifier would return a value between 0 and 1, that reflected the probability of the patients having metastases. The algorithm proposed in [13] achieved sensitivity of 90% and a specificity of 74%, while the one proposed in [14] achieved a higher specificity of 89%, keeping the same sensitivity of 90%.

Papandrianos and his team [15,16,17] have published three papers describing the work they have made on this field, devoted to the development of Convolutional Neural Networks (CNN) models for automatic classification of whole-body scans from patients with bone metastases. Just like Sadik et al., the authors intended to classify the body scans as a whole, and not the hotspots individually. In [15,16] they were dealing with a two-class classification problem regarding the presence (malignant scan) or absence (healthy scan) of bone metastases in patients with breast and prostate cancer, respectively. The best CNN architectures in [15,16] achieved an accuracy of 92.0/97.4%, a sensitivity of 94.0/96.5% and a specificity of 92.0/96.8%. The major problem with these models is that in the clinical practice the division of the bone scans into healthy or malign is oversimplified, as it ignores the fact that some patients suffer from benign conditions which will reveal several suspicious uptakes of nonmetastatic origin in the final images. As they aimed to cope with a two-class classification problem, all scans from patients containing degenerative lesions and other nonmalignant bone uptakes were removed in a manual preselection process. This is a major drawback, as a fully automatic algorithm to assess whole body scintigraphy should also be able to classify false positive bone uptakes as benign lesions. In [17] the same authors investigated a way to partial solve this problem, by developing a similar CNN based algorithm to classify bone scintigraphy images as healthy, malignant or degenerative, leading to a three-class classification problem. The best CNN architecture achieved a sensitivity of 92.7% and a specificity of 96.0%. Although the automatic distinction between malignant and nonmalignant images is an improvement over the previous models, it does not offer a solution for the cases in which one patient has bone uptakes with both malignant and nonmalignant origins, which is one of the major problems in visual bone scintigraphy assessment. In fact, neither of the papers proposed by Papandrianos et al. or Sadik et al. present an algorithm that is capable of quantifying the bone lesions individually, which is essential when an objective assessment of the disease staging is needed. It is not enough to build an algorithm that is able to distinguish images that present solely malignant lesions from those that present solely benign lesions. A suitable algorithm must be able to quantify and classify each lesion individually.

The only algorithms developed to classify bone lesions individually are the ones found in the Master theses of Dang [18] and Belcher [19]. In both works, a CNN was developed to classify hotspots in bone scintigraphy images for prostate cancer, by determining whether they had a high or low risk of being bone metastases from PCa metastatic cancer. The final CNN from [18] had an accuracy, true positive rate and AUC (Area Under the ROC Curve) of 89.0%, 98.0% and 0.96, respectively. To measure the CNN performance, [19] only used the area of the ROC curve, for which was obtained a score of 0.974. Despite appearing to be a promising approach to the classification of hotspots in bone scintigraphy images, the previously described works use supervised techniques, which rely on an extensive number of labeled data. The access to such a large data set was only possible due to EXINI Diagnostic AB, which is a Sweden based company that uses artificial intelligence to develop automated analysis platforms for medical images like cardiac, brain and bone scans [20]. It has shown to be quite popular among researchers working in the quantification of bone metastases. EXINI has developed the aBSI (automated Bone Scan Index), a software only medical device that provides a fully quantitative assessment of a patient’s skeletal disease on a bone scan, as the fraction of the total skeleton weight [21]. As it is a closed-source software, little is known about its operating principles, except that it was trained to classify hotspots as lesions using a collection of more than 40,000 hotspots derived from bone scans of patients with a variety of metastatic cancers. It is able to segment the skeleton, identify hotspots, quantify their intensity and classify them as lesions [22].), which provided them with a database composed by more than ten thousand labeled hotspots from bone scans. Such large scale annotated data sets are, however, rare in the medical context. Training a CNN from scratch to perform bone lesion classification would require thousands of labeled images, a task that would not only be extremely complex and time consuming, but also dependent on the availability of experienced physicians. Furthermore, the labelling would be subject to the subjectivity inherent in the classification of lesions detected in bone  scintigraphy.

The algorithms developed so far for the assessment of whole-body bone scans either use fully supervised learning algorithms, which require access to a (big) labeled data set, or rely on some sort of manual removal of false positives. Here, we propose a semisupervised method for the classification of automatically detected hotspots in bone scintigraphy images.

## 3. hotBSI: Semisupervised Iterative Algorithm for Hotspots Classification

The core and main contribution of the present paper is the hotBSI (hotspots on Bone Scintigraphy Images) algorithm. This algorithm was derived from the need of hotspots false positive reduction scintigraphy images, in the presence of not completely labeled database. Section 3.1 explains the workings of hotBSI, wihch can be used with any classifier of choice. The classifiers used in the present work are listed in Section 3.2.

### 3.1. hotBSI Description

An initial classifier $C_{0}$ was first trained, in the presence of noise, to distinguish between malign from nonmalign hotspots. It should be pointed out that this classifier is trained under a lot of noise, as it was assumed that every detection in a bone scan belonging the *malign* category belonged to malign class, which is not true, as the majority of the detections in these scans are actually nonmalign. The next stage involves an iterative process through the following steps:

1. The last trained classifier, $C_{i-1}$, is used to classify the detections on the scans belonging to the malign class. For each detected region, the classifier returns the likelihood that the region comes from the *malign* or *nonmalign* class;
2. For each patient in the malign category:
  (a) The detection with the highest likelihood of being malignant is selected;
  (b) All other detections with likelihood of being malignant *higher than a predetermined threshold* (if any) are also selected.
3. A new training data set is created, so that detections made on nonmalign scans are considered as false positives (and labeled as 0) and the above selected regions are considered as true-positives (or malign hotspots, labeled as 1);
4. Train a new classifier $C_{i}$ with the new training data set.

**Figure 1:**
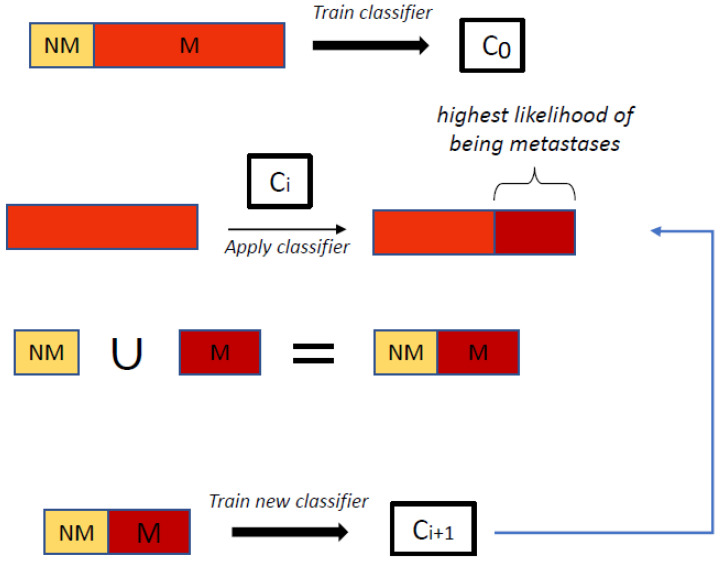
hotBSI algorithm. NM stands for detections labeled as nonmalignant, while M stands for detections labeled for training in a given iteration as malignant.

The algorithm runs during a predetermined number of iterations (set as 100 in the current experiments). Other stopping criteria will be pursued in the future. A schematic description of hotBSI is given in Algorithm 1 and Figure 1. The value of the threshold was set to 0.8.
**Algorithm 1:** hotBSI algorithm
Inputs:  NM - feature set from all the hotspots extracted from the nonmalign images  M - feature set from all the hotspots extracted from the malign images  T - threshold (default as 0.8)  NrIt - number of iterations (default as 100) Output:  C - a classifier to classify new hotspots as nonmalign or malign
 1:Train an initial classifier, $C_{0}$, with the input features (NM ∪ M) 2:**for** i = 1:NrIt **do** 3:    Empty M 4:    **for** each patient in the *malign* set **do** 5:        Use $C_{i-1}$ to predict the probabilities of the detections to be a metastases ($P_{\mathrm{met}}$) 6:        Identify the hotspot with the highest likelihood of being a metastasis ($P_{\max}$) 7:        **for** d = 1: number of detected hotspots for the current patient **do** 8:           **if** $P_{\mathrm{met}}\left(d\right)==P_{\max}$ || $P_{\mathrm{met}}\left(d\right)>T$ **then** 9:               Add the hotspot to M10:    Create a new training set, NM ∪ M11:    Train a new classifier $C_{i}$ with the new training data set12:**return**$C_{NrIt}$


### 3.2. Learning Algorithms

The classifiers were trained using four different supervised learning algorithms: (i) a support vector machine (SVM) trained with a linear kernel with scale 1, where the values obtained with the linear SVM score function (bias = 1.08) were transformed into posterior probabilities using the sigmoid function with slope −1.40 and intercept 0.06; (ii) *k*-nearest neighbors (KNN), trained with five nearest neighbors with uniform weighting and the Euclidean distance function as the distance metric; (iii) decision trees (DTs), trained with a minimum of 10 samples per branch node, a maximum number of splits equal to the number of samples minus one and the Gini’s diversity index as the split criterion and (iv) linear discriminant analysis (LDA) with ’Delta’ (linear coefficient threshold) and ’Gamma’ (amount of regularization) both equal to 0.

## 4. Materials and Methods

This section encompasses several details related with the implementation and evaluation. The database is described in Section 4.1. The methodology is given in Section 4.2, including the method for the detection of hotspots, the list of extracted features, and the state of the art classification techniques used for comparison with the proposed iterative method. Lastly, the evaluation methodology is presented in Section 4.3.

### 4.1. Database

The database consists of 195 bone scintigraphy images from 102 patients with prostate cancer with suspected bone metastatic disease. The equipment used for scanning patients was either a *Millennium MG* (GE Medical Systems), which digitally record anterior and posterior scans with a resolution of 1024×256 pixels, or a *BrightView* (Philips Healthcare), which digitally records anterior and posterior scans with a resolution of 1024×512 pixels. The pixel depth (maximum number of counts which could be stored in a pixel) is 16-bits for every image. For each bone scan, a medical report describing the condition of the patient in question written by a nuclear medicine physician is available. All data was provided by Instituto Português de Oncologia do Porto Francisco Gentil (IPO Porto). The data was collected and held anonymously and the developed algorithms did not contain information concerning the patients, but rather information extracted from the data during the algorithm development. This project was authorized by IPO-Porto Healthcare Ethics Committee.

The scans were organized into three categories: (i) *healthy*, if no suspicious bone uptake was detected, (ii) *benign*, if bone hotspots with no metastatic origin are present or (iii) *malign*, if bone metastases exist. Table 1 summarizes the available database, including the number of bone scans per category. It is important to point out that images from the malign category can also present benign hotspots.

### 4.2. Methodology

**Figure 2:**
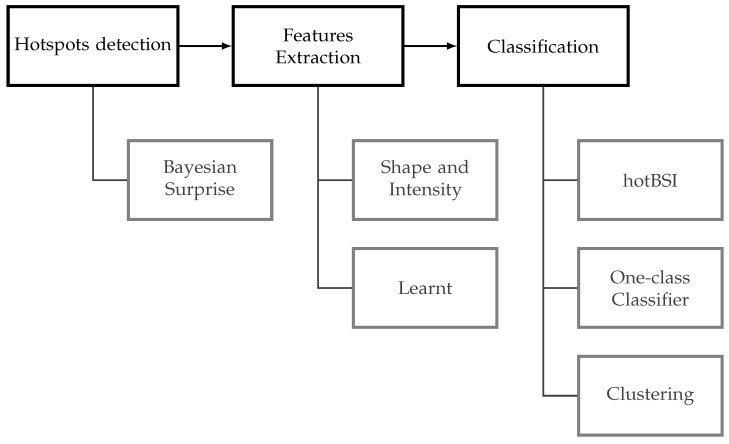
Methodology overview.

The methodology proposed in this paper for the automatic false positives reduction of hotspots in bone scintigraphy images involves a three step process (Figure 2): detection of the hotspots, extraction of features from the detected hotspots and training an algorithm for the classification of the detected regions.

#### 4.2.1. Hotspots Detection

Although a customised hotspots detection algorithm was developed, we note that the here proposed algorithm, hotBSI, is independent of the detection algorithm and can be used with any detector of choice.

**Figure 3:**
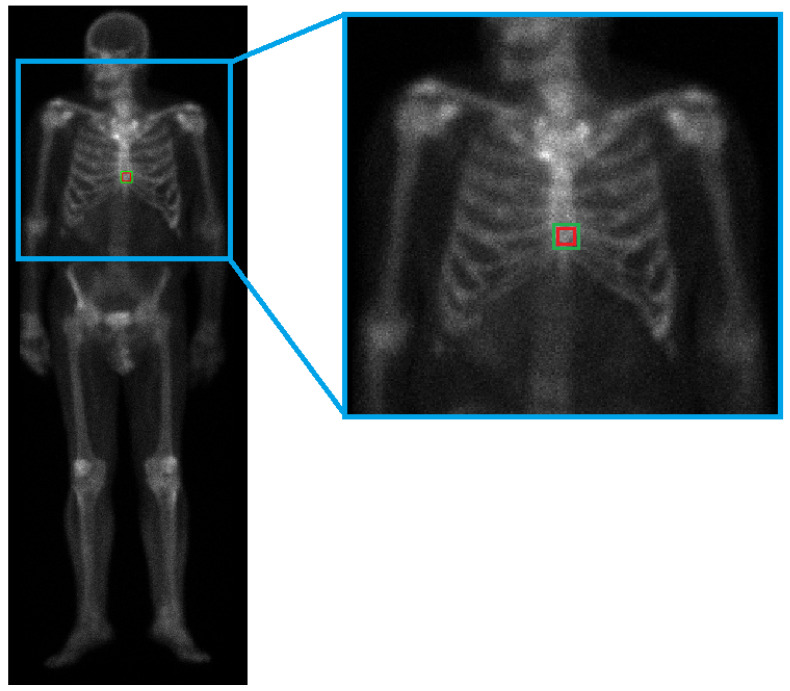
Detection illustration. Note that for illustration purposes, only one region is being tested in the current image. In the full detection algorithm, all of the regions within the mask are evaluated.

The present detector is based on the approach proposed in [23], where a technique based on Bayesian surprise is used to detect calcifications in mammogram images. The algorithm takes advantage of the fact that the hotspots are bright regions (that is, regions with higher grey levels) surrounded by pixels with lower grey values. The first step of the algorithm consists in applying a mask to the original image to exclude the background and keep solely the body of the patient. The mask was obtained by binarizing the original grayscale image by thresholding using the Otsu’s method [24] Then, the hotspots were detected through the following steps (Figure 3):

1. Consider a square patch of the masked image with half-radius $r_{\mathrm{in}}$;
2. Consider the region surrounding the patch described in 1, defined by a radius $r_{\mathrm{out}}=\sqrt{2}\;\cdot \;r_{\mathrm{in}}$ and with centre coinciding with that of the inner patch;
3. Calculate the mean grey level of both the inner patch and the surrounding region;
4. Compare the mean grey levels: if the absolute difference of the two values is higher than a certain threshold δ, the inner patch is considered a hotspot.

The steps were repeated for every patch in the masked image with the following empirically obtained values: $r_{\mathrm{in}}=5\;cm$ and δ=20. The final threshold δ was chosen to obtain as few false positives as possible, while at the same time not losing any malignant hotspot. In this way, a considerable amount of hotspots not related to bone metastases are detected with this algorithm. These hotspots can be due to some kind of benign bone condition or can be due to normal and healthy physiological processes. Since the patient condition is determined through the assessment of the malign bone lesions, the number of false positive detections should be reduced. This was achieved through the development of classification algorithms, which require the extraction of features from the detected regions.

#### 4.2.2. Feature Extraction

The detection algorithm is followed by a feature extraction stage which obtains the features from the hotspots that will serve as input to a classification algorithm. Two types of features were extracted: handcrafted low-level features and learnt high-level features.

Sixteen (16) shape and four (4) intensity handcrafted features were first extracted from each automatically detected region. The list of the handcrafted features can be found in Table A8 in the Appendix A.

**Figure A1:**
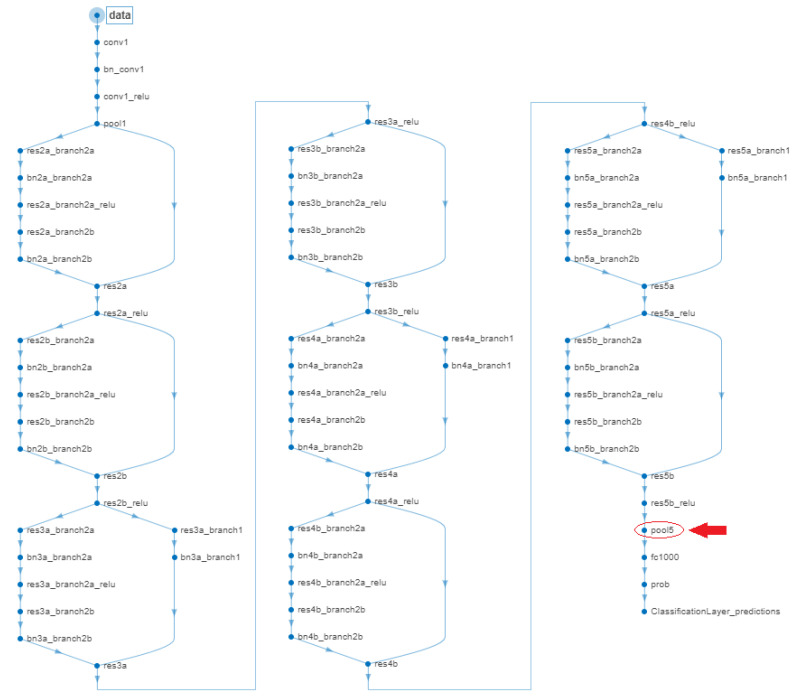
Diagram of ResNet18 with highlighted “pool5” layer.

High-level features were extracted using the convolutional base of a pretrained CNN. Since the used CNN requires input images of size n×n×3, each automatically detected patch was converted into RGB by replicating the grey image in each channel. The detections were also resized so that their size matched the one required by the input layer of the network in question, 224×224×3. Next, a pretrained ResNet18 network was used to extract features from the regions (we refer to [25] for a review of deep learning). The “pool5” layer was used as the output layer to extract a 512-dimensional vector for each possible hotspot (see Figure A1 of Appendix B).

#### 4.2.3. Methods Used for Comparison

Two state-of-the-art methods were used as a comparison with the hotBSI algorithm here proposed. Given the lack of a fully annotated database, which precluded the use of supervised learning methods, an unsupervised and a semisupervised learning algorithms were used.

For the unsupervised method, a clustering technique with the *k*-means clustering algorithm was used. A *k*-means clustering algorithm with two clusters was initially applied to the training set, and a model for the classification of new data was built by assuming that each final cluster represented a class and by assigning each hotspot from the test set to the nearest cluster centroid. By choosing two clusters, it was expected that the data could be partitioned into a cluster of nonmalign data and a cluster of malign data (metastases). The distance metric used for defining the initial clusters, as well as to assign new data to these clusters, was the square Euclidean distance.

The semisupervised method was a one-class classification (OCC) algorithm. The hotspots extracted from the nonmalign set (false positives) were used to train an one-class support vector machine algorithm (OC-SVM), and a model which classified new hotspots as nonmalign or as outliers (here considered to be metastases—true positives) was obtained. The OC-SVM algorithm used was the one proposed by [26] and was trained with an outlier fraction of 5%, a Gaussian kernel function with a Kernel scale parameter of 1.81 and a Sequential Minimal Optimization (SMO) as an optimization routine.

### 4.3. Evaluation Methodology

In the present work, detections automatically made in scans from the bone scan category *Healthy* and *Benign* were considered as false positives, whereas detections extracted made in scans with the bone scan category *Malign* were considered as true positives.

A test set was created with the detections extracted from scans of 30 patients randomly chosen from the *Healthy*, *Benign* and *Malign* bone scan categories (10 patients per category). This test data set (and only this test data set) was manually labeled, identifying the true detections (malign) and the false positive ones (nonmalign). The number of patients and detections per class for the training and the test set are presented in Table 2. We do acknowledge the imbalanced nature of the data set and intend to experiment on ways to deal with this issue in the future [27,28].

The algorithms were evaluated using common performance metrics such as sensitivity, specificity, accuracy, precision, false positive rate (FPR), F1-score and AUC (area under the ROC curve). In addiction, the false negative rate (FNR) is also calculated, as it was considered that a low FNR was of special importance for this particular classifier.

Since the goal of this algorithm is to be used in the clinical practice to aid physicians in the diagnose and follow-up of patients with metastatic cancer, it is important that the final algorithm has a FNR as low as possible. A high FNR would mean that the algorithm was classifying a lot of malign hotspots as nonmalign, which could be dangerous to the patient, as it was failing to diagnose them with the disease and preventing them from having access to an early treatment.

## 5. Results

In this section, the results are reported. The performance of the detection algorithm is firstly shown (Section 5.1), followed by the analysis of the efficiency of the different classification algorithms to remove false positive detections (Section 5.2). For each classification model, the results obtained when using both handcrafted and high-level features are presented.

### 5.1. Detection Results

**Figure 4:**
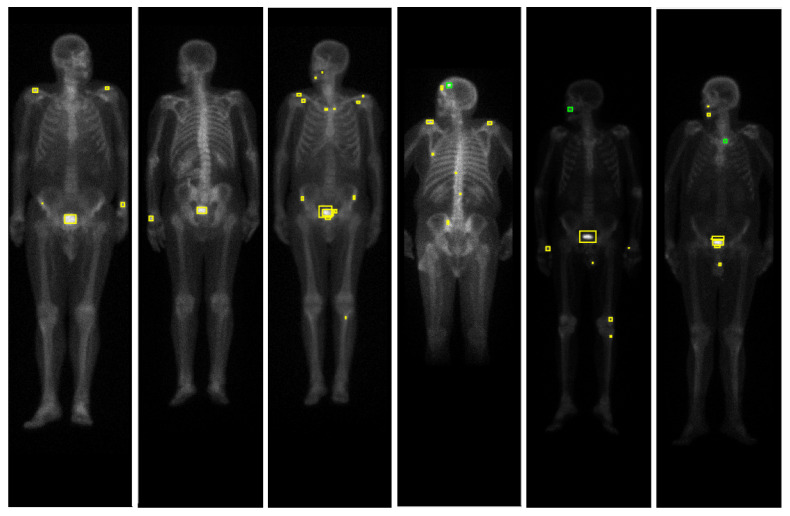
Results of the detection algorithm in bone scintigraphy images from the nonmalign set. The colours of the bounding boxes were manually chosen for the purposes of illustration, according to the respective medical report of the patient: red represents metastases, green represents benign bone lesions and yellow represents false positives (hotspots that are neither malign nor benign lesions).

**Figure 5:**
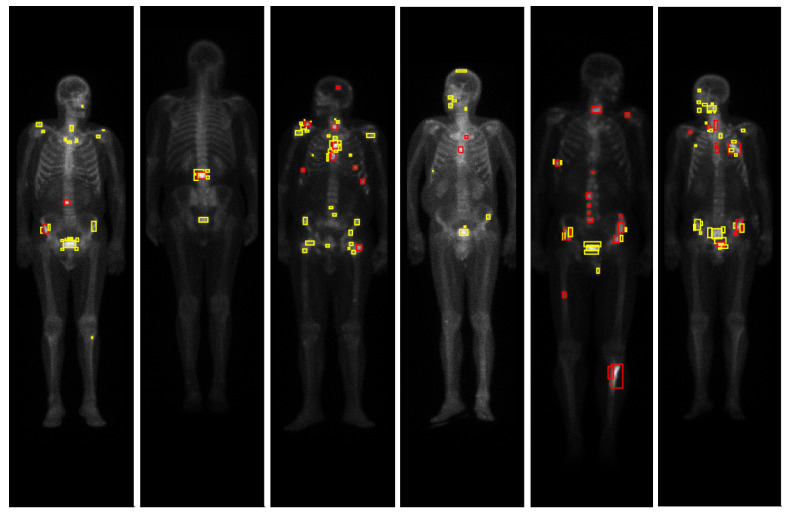
Results of the detection algorithm in bone scintigraphy images from the malign set. The colours of the bounding boxes were manually chosen for the purposes of illustration, according to the respective medical report of the patient: red represents metastases, green represents benign bone lesions and yellow represents false positives (hotspots that are neither malign nor benign lesions).

The algorithm described in Section 4.2.1 successfully detected all the hotspots corresponding to metastases (see Table 3). Figure 4 illustrates the detection algorithm in bone scintigraphy images from the nonmalign set, while Figure 5 illustrates the detection algorithm in bone scintigraphy images from the malign set. Comparing the results with the respective patient’s medical reports, it can be concluded that the algorithm successfully detected all the hotspots corresponding to metastases. On the other hand, this algorithm presents a high rate of false positive detections: approximately 73% of the detected hotspots were not metastases. Observing the figures, it can be seen that most of the detected hotspots are healthy or benign (that is, nonmalign), while only a small percentage of the detected hotspots are actually metastases.

### 5.2. False Positive Attenuation Results

The proposed algorithm, hotBSI, was used to classify the hotspots from the test set. Table 4 and Table 5 gather the performance results for the hotBSI trained with SVM/KNN and DTs/DLA, respectively. Results obtained with the *k*-means and one-class classification algorithms are shown in Table 6. In all tables, results for both handcrafted (HC) and ResNet18 (RN18) features are presented.

**Figure A2:**
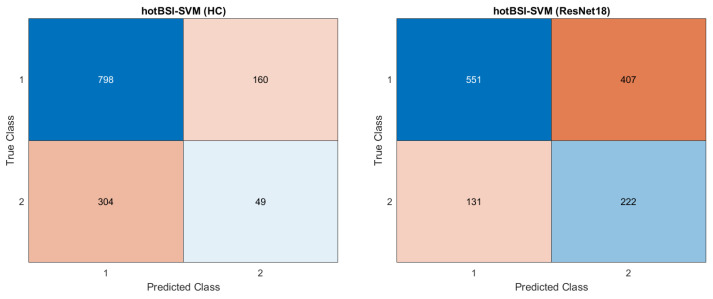
Confusion matrices for the hotBSI-SVM trained with handcrafted (**left**) and ResNet18 (**right**) features.

**Figure A3:**
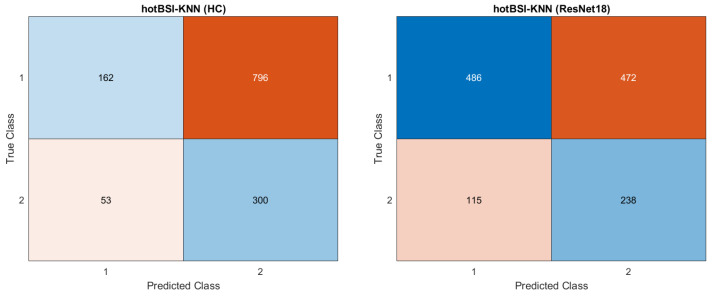
Confusion matrices for the hotBSI-KNN trained with handcrafted (**left**) and ResNet18 (**right**) features.

**Figure A4:**
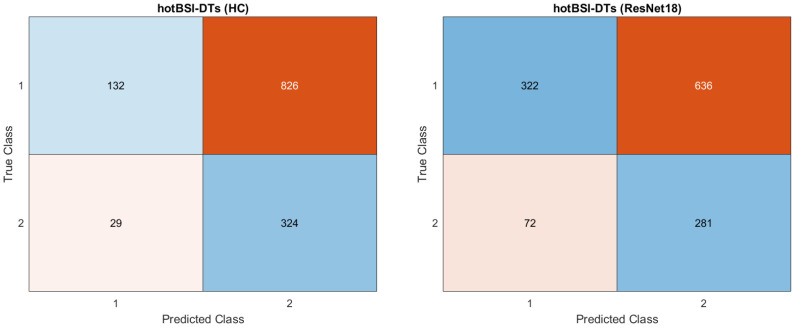
Confusion matrices for the hotBSI-DTs trained with handcrafted (**left**) and ResNet18 (**right**) features.

**Figure A5:**
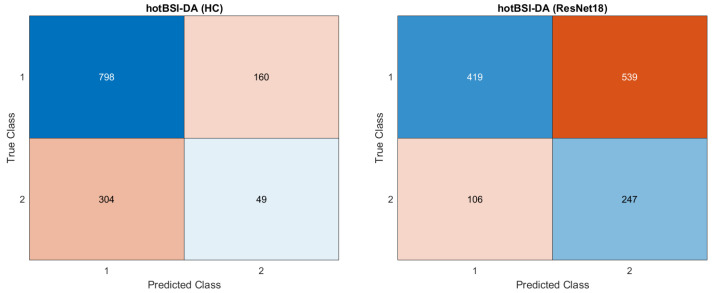
Confusion matrices for the hotBSI-DA trained with handcrafted (**left**) and ResNet18 (**right**) features.

**Figure A6:**
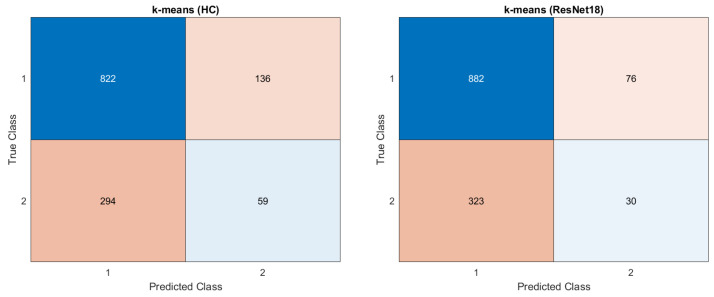
Confusion matrices for the k-means algorithm trained with handcrafted (**left**) and ResNet18 (**right**) features.

**Figure A7:**
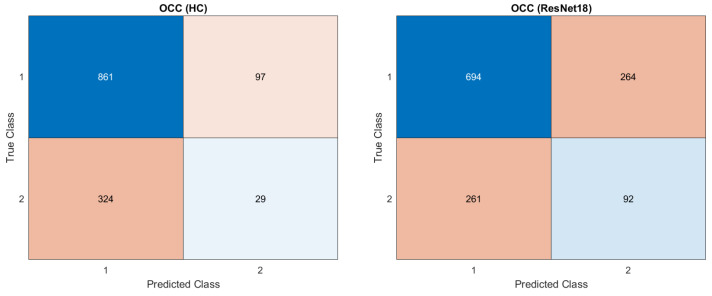
Confusion matrices for the OCC algorithm trained with handcrafted (**left**) and ResNet18 (**right**) features.

The confusion matrices obtained with all the algorithms can be found in Figure A2,Figure A3,Figure A4,Figure A5,Figure A6,Figure A7 of Appendix C.

## 6. Discussion

This work had as main goal the development of an algorithm capable of automatically identifying metastases in bone scintigraphy images from patients with prostate cancer. If successful, this algorithm could be used in the clinical practice to quantify bone scans and work as an aiding tool for the diagnosis and follow-up of patients with bone metastases. Despite consensus on the need for such an algorithm, and despite efforts of the scientific community to develop one, such a diagnosis tool is currently unavailable in the medical community.

The current work differs from the ones developed so far in the same topic in the sense that it does not resort to a fully supervised data set to train the classifier. An algorithm that proves to be successful even without the access to a labeled data set can be extremely useful in the clinical context, where access to a labeled database is often difficult to achieve.

Here, an algorithm for the automatic detection of hotspots in bone scans was suggested, followed by the development of an algorithm capable of classifying the detected hotspots as malign or nonmalign. The detection algorithm proved to be successful on a database of patients with prostate cancer, as all malign hotspots were correctly identified. This was guaranteed by choosing a threshold value that would ensure that no metastases candidates were left undetected. This came at the cost of a high false positive detection rate, meaning that most of the hotspots detected by the algorithm were nonmalign. As the patient condition is determined through the assessment of the malign bone lesions, an algorithm for the attenuation of the false positive was developed. The evaluation metrics considered the most relevant for the current classifier and the respective values obtained for the proposed algorithm are now discussed.

### 6.1. Area under the ROC Curve (AUC)

The AUC values, usually close or equal to 0.50, translate the low to none capacity of most classifiers to distinguish between nonmalign and malign hotspots. The highest AUC score was obtained with the hotBSI trained with SVM and ResNet18 features (AUC = 0.66).

### 6.2. Sensitivity and Specificity

High values of sensitivity and specificity were only obtained when the classifier was biased toward one class: high sensitivity scores (>0.85) were always accompanied by a low specificity score, which meant that it was considering almost every hotspot to belong to the positive (malign) class; on the other hand, high specificity scores (>0.85) were always accompanied by a low sensitivity score, meaning that it was assigning the majority of hotspots to the negative (nonmalign) class. Neither situation is desirable for the final algorithm. The classifiers with more balanced scores in terms of sensitivity and specificity were (i) the hotBSI trained with SVM and ResNet18 features (sensitivity = 0.63, specificity = 0.58) and (ii) the hotBSI trained with KNN and ResNet18 features (sensitivity = 0.67, specificity = 0.51).

### 6.3. False Negative Rate (FNR)

An important evaluation metric for an algorithm whose goal is to classify hotspots in patients who might have bone metastases is the false negative rate. It is desirable that this value is as low as possible, as a low FNR would mean that the classifier was incorrectly labelling a lot of malign hotspots (metastases) as nonmalign; this would result in an algorithm that would label patients with metastatic cancer as healthy, which would be dangerous is the clinical context. Very low FNR only happened with classifiers that were assigning almost every hotspot to the malign class: taking a look at the hotBSI trained with decision trees it can be observed that a FNR rate of 0.08 was obtained. Although at first glance this may seem like an almost perfect result, further analysis on the remaining metrics lead us to conclude that this FNR only happens because the classifier is assigning almost every hotspot to the malign class and, therefore, it had a low probability of missing metastases (sensitivity = 0.92, specificity = 0.14). Such a classifier is obviously not acceptable, as it has no discriminatory power. Classifiers that obtained lower FNR while keeping more acceptable values for the other metrics include (i) the hotBSI trained with discriminant analysis and ResNet18 features (FNR = 0.30), (ii) the hotBSI trained with KNN and ResNet18 features (FNR = 0.33) and (iii) the hotBSI trained with SVM and ResNet18 features (FNR = 0.37).

### 6.4. *False Positive Rate Reduction*

As mentioned in Section 5.1, the detection algorithm presented a false positive rate of 73.07%. After applying the classifiers to these detections, the lowest FPR scores were obtained with (i) the hotBSI trained with SVM and handcrafted features (FPR = 0.18), (ii) the OCC trained with handcrafted and ResNet18 features (FPR = 0.10 and FPR = 0.28, respectively) and (iii) *k*-means with handcrafted features (FPR = 0.14). This low values are, however, only due the fact that these algorithms were classifying most of the metastases as nonmalign, which is not desirable, as it will lead to a high FNR. The classifier that presented the lowest FPR while keeping an acceptable value for the FNR was the hotBSI trained with SVM and ResNet18 features (FPR = 0.42). This represents a decrease of 30.59% compared to the FPR score obtained with initial detection algorithm, when no classifiers had been yet applied.

### 6.5. Comparison with the State-of-the-Art Algorithms

Table 7 gathers the best results obtained with the hotBSI algorithm, as well as the best results obtained with the *k*-means and one-class classifier. The best hotBSI algorithm was considered to be the one trained with SVM and ResNet18 features; the best *k*-means and one-class algorithms were considered to be the ones trained with handcrafted and ResNet18 features, respectively. The proposed algorithm shows superiority in almost every metric, in particular in the AUC (0.66 compared to 0.50 from the OCC classifier), sensitivity (0.63 compared with 0.17 and 0.26 from the *k*-means and OCC classifiers, respectively) and the false negative rate (0.37 compared with 0.83 and 0.74 from the *k*-means and OCC classifiers, respectively). It should be noted that the only two metrics in which the state-of-the-art algorithms performed better were accuracy and specificity. This is clearly explained by noting that this happens since these algorithms are classifying most of the hotspots as nonmalign (note the low sensitivity from the same classifiers); as a consequence, they will present a high specificity, as if most of the hotspots are being classified as nonmalign there is a better chance that the algorithm will correctly classify nonmalign hotspots as nonmalign. Besides the low specificity, this comes with a cost of a high false negative rate, as a lot of malign hotspots are being incorrectly classified as nonmalign. The better scores in accuracy are also easily explained by looking at the percentage of nonmalign and malign hotspots present in the test set: 73% of these hotspots were from the nonmalign category, while only 27% were from the malign category. Because the *k*-means and OCC classifiers are manly assigning hotspots to the negative (nonmalign) class, and because most of the test set is composed by hotspots from this class, they will get a high accuracy score, even if most of the data is wrongly classified. Having all of this into account, it can be concluded that the proposed algorithm performs better than the state-of-the-art algorithms at the task of hotspots classification and, therefore, at the task of false positive attenuation.

## 7. Conclusions

An algorithm for the classification of automatically detected hotspots in bone scintigraphy images of patients with prostate cancer was proposed. Such an algorithm can be used in combination with computer-assisted PCa detection approaches such as the one described in [29], making it extremely useful in the medical community, as it provides the physicians with an aiding tool to quantify whole-body bone scans from patients with bone metastases.

The biggest challenge when building such an algorithm is the lack of a labeled data set. Here, we tried to overcome that problem by developing an algorithm that only requires knowledge about the type of bone scan from which the hotspot is extracted from. Comparison with state-of-the-art algorithms shows superiority of the proposed method. However, analysis of the performance metrics obtained for the hotBSI shows that this algorithm is still not ready to be used in the clinical practice: the not so high scores for sensitivity, specificity and AUC are still a concern; the false negative rate, despite clearly inferior to the state-of-the-art algorithms, is also still high. Improvements on the algorithm are therefore need. These include:

- Finding features that are more discriminative, for instance, by using a different pretrained network, by extracting features from different layers or by extracting features from autoencoders;
- Using other classifiers to train the hotBSI;
- Apply variations in the hotBSI, for example, by choosing a stopping criteria in the iteration that is not the number of iterations;
- Retrain the algorithm with a more balanced data set.

Once an algorithm with a performance that is considered good enough to be used in the clinical practice is obtained, a quantitative image biomarker can be used to automatically quantify a bone scintigraphy of new patients with prostate cancer. Literature shows that the most adequate image biomarker for quantifying a bone scan is the Bone Scan Index (BSI) [22,30,31,32,33].

The final goal is to build a software that can be used in the clinical context, that is capable of not only quantifying a given bone scintigraphy of a patient with prostate cancer, but also give information about disease progression, response to treatment and disease prognosis. Such a software will make the process of assessing a bone scan more objective, simpler and faster, and will for sure be an asset in the medical community.

## Figures and Tables

**Table 1:** Database summary. The database consists of a total of 195 bone scans divided into one of three categories: healthy, if no suspicious bone uptakes were detected, benign if bone hotspots with benign origins are present, or malign, if the images have bone metastases.

Bone Scan Type	No of Bone Scans
Healthy	37
Benign	72
Malign	86
**Total**	**195**

**Table 2:** Split of the dataset.

Bone Scan Category	No. of Patients		No. of Detections	
	Training	Test	Training	Test
Nonmalign	89	20	1941	393
Malign	76	10	5620	918
**Total**	**65**	**30**	**7561**	**1311**

**Table 3:** Results of the detection phase.

TP	1.00
FN	0.00
FP	0.73
Sensitivity	0.00
FNR	0.00
Precision	0.58
F1	0.73
FPPI	32

**Table 4:** Results with the hotBSI trained with support vector machine and *k*-nearest neighbors.

Classifier	SVM		KNN	
	HC	RN18	HC	RN18
Sensitivity	0.13	0.63	0.85	0.67
Specificity	0.83	0.58	0.17	0.51
Accuracy	0.65	0.59	0.35	0.55
FNR	0.86	0.37	0.15	0.32
FPR	0.18	0.42	0.83	0.49
Precision	0.23	0.35	0.27	0.34
F1	0.17	0.46	0.41	0.45
AUC	0.50	0.66	0.52	0.62

**Table 5:** Results with the hotBSI trained with decision trees and linear discriminant analysis.

Classifier	DTs		LDA	
	HC	RN18	HC	RN18
Sensitivity	0.92	0.80	0.83	0.70
Specificity	0.14	0.33	0.19	0.43
Accuracy	0.35	0.46	0.36	0.51
FNR	0.08	0.20	0.17	0.30
FPR	0.86	0.66	0.81	0.56
Precision	0.28	0.31	0.28	0.31
F1	0.43	0.44	0.41	0.43
AUC	0.46	0.57	0.44	0.59

**Table 6:** Results with OCC and Kmeans.

Classifier	*k*-Means		OCC	
	HC	RN18	HC	RN18
Sensitivity	0.17	0.08	0.08	0.26
Specificity	0.86	0.92	0.90	0.72
Accuracy	0.67	0.70	0.68	0.60
FNR	0.83	0.92	0.92	0.74
FPR	0.14	0.92	0.10	0.28
Precision	0.30	0.28	0.23	0.26
F1	0.22	0.13	0.12	0.14
AUC	–	–	0.51	0.50

**Table 7:** Comparison of the best hotBSI with the best state-of-the-art algorithms.

	hotBSI (RN18)	*k*-Means (HC)	OCC (RN18)
Sensitivity	0.63	0.17	0.26
Specificity	0.58	0.86	0.72
Accuracy	0.59	0.67	0.60
FNR	0.37	0.83	0.74
FPR	0.42	0.14	0.28
Precision	0.35	0.30	0.26
F1	0.46	0.22	0.14
AUC	0.66	–	0.50

## Appendix A. Handcrafted Features

The full list of handcrafted features is given in Table A1.

**Table A1:** Name and description of the handcrafted features. Top part of the table corresponds to shape measurements, while the bottom half are pixel value measurements.

Property	Description
Area	No. of pixels in the region
AxisLengthRatio	Ratio between *MajoraxisLength* and *MinoraxisLength*
BoundingBox	Position and size of the smallest box containing the region
Centroid	Center of mass of the region
ConvexArea	Number of pixels in ConvexImage ${}^{1}$
Eccentricity	Eccentricity of the ellipse ϵ ${}^{2}$
EquivDiameter	Diameter of a circle with the same area as the region
EulerNumber	No. of objects in the region minus the no. of holes in those objects
Extent	Ratio of pixels in the region to pixels in the total bounding box
FilledArea	Number of on pixels in FilledImage ${}^{3}$
InvCircularity	Inverse of the roundness ${}^{4}$ of the object
MajoraxisLength	Length (in pixels) of the major axis of $\hat{\epsilon }$ ${}^{5}$
MinoraxisLength	Length (in pixels) of the minor axis of $\hat{\epsilon }$
Orientation	Angle between the *x*-axis and the major axis of $\hat{\epsilon }$
Perimeter	Distance around the boundary of the region
Solidity	Proportion of the pixels in the convex hull that are also in the region
MaxIntensity	Value of the pixel with the greatest intensity in the region
MeanIntensity	Mean of all the intensity values in the region
MinIntensity	Value of the pixel with the lowest intensity in the region
WeightedCentroid	Center of the region based on location and intensity value

${}^{1}$*ConvexImage*: Image that specifies the ConvexHull${}^{6}$, with all pixels within the hull filled in (binary image). ${}^{2}$ϵ: ellipse that has the same second-moments as the region. ${}^{3}$*FilledImage* Image the same size as the bounding box of the region, returned as a binary. ${}^{4}$ The roundness of an object is defined as $\frac{4\;\cdot \;\mathrm{Area}\;\cdot \;\pi }{{\mathrm{Perimeter}}^{2}}$. ${}^{5}$ $\hat{\epsilon }$: ellipse that has the same normalized second central moments as the region. ${}^{6}$ *ConvexHull*: Smallest convex polygon that can contain the region.

## Appendix B. Learnt Features

A schematic representation of the architecture of the ResNet18 network is given in Figure A1.

## Appendix C. Confusion Matrixes

The confusion matrices obtained during the false positive attenuation phase (Section 5.2) are given in Figure A2,Figure A3,Figure A4,Figure A5,Figure A6,Figure A7.
